# Teaching lessons learnt by civil-engineering teachers from the COVID-19 pandemic at the University of Burgos, Spain

**DOI:** 10.1371/journal.pone.0279313

**Published:** 2022-12-16

**Authors:** Víctor Revilla-Cuesta, Marta Skaf, Ana B. Espinosa, Vanesa Ortega-López

**Affiliations:** 1 Department of Civil Engineering, Escuela Politécnica Superior, University of Burgos, Burgos, Spain; 2 Department of Construction, Escuela Politécnica Superior, University of Burgos, Burgos, Spain; Fondazione Ugo Bordoni, ITALY

## Abstract

The COVID-19 lockdown in Spain caused abrupt changes for students following the Bachelor’s Degree in Civil Engineering at the University of Burgos when face-to-face classes switched to online teaching. The recovery of face-to-face teaching after lockdown meant that classes were taught with obligatory social distancing and the use of masks. Teachers were therefore unable to interact with students closely, to perceive their facial expressions during class, or to conduct group work. The changes to civil-engineering teaching linked to the COVID-19 pandemic and the lessons that civil-engineering teachers learnt from the new teaching scenarios are studied in this paper. The reflections of teachers throughout all three stages of the pandemic (pre-pandemic and lockdown, during lockdown, and post-lockdown), and the qualitative and mixed analysis of their responses to a survey of open-ended questions contributed to the identification of six major lessons: (1) asking questions and using real-time quiz tools enliven classes and help to determine which concepts to emphasize for proper student understanding; (2) autonomous student learning can be promoted through the provision of supplementary documentation and the digitalization of solutions to classroom exercises; (3) virtual site visits and real visual examples interspersed with explanations bring concepts closer to their real applications; (4) the delivery of projects in the form of audio-recorded presentations enable their distribution, so that other students can also learn from them as well as the students who created them; (5) online videoconferences, adapted to the concepts that are addressed, facilitate fast and flexible communication with students; and (6) online continuous-assessment exams can promote better student learning patterns and final-exam preparation. Nevertheless, these six lessons were drawn from the experience of teachers at a small Spanish university where the period of solely online teaching during the COVID-19 pandemic lasted only four months. Thus, it would be interesting to analyze the experience of civil-engineering teachers at larger universities and universities that had longer periods of solely online teaching. A study of the level of implementation of the six aspects when the pandemic is declared over might also be worthwhile.

## 1. Introduction

The COVID-19 pandemic, which broke out towards the end of 2019, has had a major impact on our way of life. Some of the most foreboding moments coincided with the outbreak of the pandemic and ignorance of the symptoms. A strict lockdown was imposed in numerous countries around the globe [1]. Post-lockdown, a series of changes to social life had the aim of drastically reducing social contact, in order to prevent the spread of the virus and, with it, the increase in contagion and deaths [2]. On the one hand, strict limits were imposed in many places of leisure, commerce, work, and education, restricting the numbers of people who could gather together in one place at any one time [3]. On the other hand, work, social, and family gatherings were curtailed, as the higher the number of interpersonal encounters, the greater the likelihood of infection [4]. These measures, together with optimum ventilation and the rigorous use of face masks, partially mitigated indoor contagion where the contagion risks were much higher than in the street [1]. However, these measures were not sufficient in many countries and, throughout the pandemic, the closure of stores and places of entertainment, and even new lockdowns, were common [5]. Furthermore, applying these health-safety measures in different sectors, such as civil engineering, led to delays in the completion of work, insufficient workforce or legal issues [6]. At present, the health situation is improving thanks to vaccination and greater immunity of the population, which has led to the gradual relaxation of these restrictions, although the precautionary principle must still prevail in social behavior [7].

Education is a social activity *par excellence* in which interpersonal contact is essential, regardless of the (face-to-face, online, dual) teaching methodology in use, as all teaching is based on teachers transmitting knowledge to students [8]. The limitations on social life due to the COVID-19 pandemic have also led to a modification of traditional patterns of teaching, in general [9], and of teaching in engineering, in particular [10]. Three major teaching phases may be distinguished, coinciding with the stages of the COVID-19 pandemic: pre-pandemic, during the initial lockdown, and post-lockdown [11].

Before COVID-19 took hold, teaching in engineering was mainly characterized by face-to-face classes taught through lectures [12] where the teacher explained the theory or exercises, while the students took the notes that they considered necessary. Subsequently, the student’s task was to study those notes, so as to prepare for the exams that they had to pass to graduate from the course. It is a style of teaching that encourages passive learning [13], as students may often assume that their task is only to study, so that they can successfully answer the exam questions [14]. The implementation of the European Higher Education Area (EHEA) partially reformed this trend, as it emphasized class attendance and continuous assessment (mid-term exams, projects, class expositions…) in the final grade of the courses [15]. In addition, the relevance of continuous assessment slowly led to the implementation of teaching methodologies that had not been very common in the field of engineering [16], such as formative assessment, cooperative learning and the flipped classroom [17]. The aim is at all times for students to participate actively in their own learning and for their active participation to be reflected in the final grade of the course [18].

The COVID-19 pandemic and the accompanying lockdown completely changed teaching patterns, following the removal of face-to-face teaching. Thus, both teachers and students had to adapt abruptly and unexpectedly to online teaching [19]. Many engineering teachers replaced face-to-face explanations with asynchronous videos and presentations for students to view online at will [20], thus allowing a better work-life balance for all involved [21]. Other teachers opted to continue teaching classes in real time, using computer tools such as Zoom, Microsoft Teams, and Skype [22]. This type of tool also became a common instrument for out-of-class teacher-student communication [23]. Favorable student opinions were reported in studies on teaching during lockdown, characterized fundamentally by empathetic attitudes towards the challenge that teachers faced when adapting to a completely new teaching methodology without any warning or preparation [19]. There is also no doubt that this period of lockdown provided the teachers with a learning experience on how to teach online, which was useful during later phases of the pandemic [24].

After lockdown, face-to-face teaching was mostly restored with a few exceptions. On the one hand, teaching continued to be conducted online in some countries, as it was considered safer from the point of view of public health [25]. On the other hand, it was necessary to perform brief localized lockdowns of the population in several countries whenever variants of the virus peaked, which meant a return to online teaching for certain periods of time [26]. However, post-lockdown teaching was mainly characterized by greater normalization of the situation, returning to face-to-face teaching in engineering [27], although it was sometimes necessary to give the class simultaneously in person and online (hybrid teaching modality), so that students who were either confined due to contagion or to close contact, could also log on to receive it [28]. The projects to be done in groups by students were also limited for sanitary reasons [29], which in turn also led to a reduction in the use of teaching methodologies that seek more active participation among students in their learning [30]. However, learning to use online communication tools, both during and after lockdown, permitted the teachers to continue tutoring students and even to continue using teaching methods such as the flipped classroom [31]. Thus, post-lockdown teaching mainly consisted of face-to-face teaching with the support of online tools.

From the teaching perspective, civil engineering is characterized by the great variety of both theoretical and practical concepts that students must learn, such as structural calculation, hydraulics, transport and logistics, project management and budgeting, team management, legislation, and economics [32, 33]. It has undoubtedly meant that the changes to teaching following the COVID-19 pandemic have been especially difficult to implement in civil engineering [34], due to the large number of concepts to be addressed [35]. Furthermore, one of the most highly valued aspects among students of civil engineering is the exemplification of the aspects addressed in class through real cases [36] or the teacher’s own professional experience [37], an aspect greatly affected by the limitation of social contact due to COVID-19 [38]. Civil-engineering teachers are experts in this engineering field who transmit their knowledge to future engineers [39]. Therefore, analyzing their experiences during the COVID-19 pandemic enables to determine aspects for the improvement of teaching in this type of courses.

For the addressed reasons, the way that teaching was performed on the Bachelor’s Degree in Civil Engineering at the University of Burgos, Spain during the three stages of COVID-19 pandemic was analyzed. The opinions collected from a representative sample of teachers of this university degree were analyzed, which shed light on teaching during the COVID-19 pandemic and helped to determine the lessons learned in relation to how to approach the teaching of civil engineering and successfully improve it after the pandemic-related restrictions had been eased. Thus, the main contribution of this paper to the corpus of teaching knowledge is the identification of aspects that led to innovative improvements for civil-engineering teaching during the COVID-19 pandemic, which could also be applied under normal conditions. The generic approach that was adopted meant that the teaching innovations could be applied to courses of different civil-engineering fields.

## 2. Materials and methods

### 2.1. Teaching framework during the COVID-19 pandemic

Teaching at the University of Burgos on the Bachelor’s Degree in Civil Engineering differed during each stage of the COVID-19 pandemic, which is the period under study, from the end of 2019 to the end of May 2022:

- Although the first news on the appearance of COVID-19 dates from the end of 2019 [24], the virus took some time to spread, so teaching was held face-to-face until mid-March 2020 (2019/2020 academic year). Thus, the teachers taught the classes with each course divided into two blocks, theoretical and practical, with exactly the same number of face-to-face teaching hours per week.
- The spread of the virus led the University of Burgos to decree the suspension of on-site classes of all its Bachelor’s and Master’s Degrees, including the Bachelor’s Degree in Civil Engineering, on March 12, 2020. On March 15, 2020, the Government of Spain decreed a strict confinement of the population, only allowing people to leave home to work when teleworking was not possible, and to do the shopping. Thus, from March 12, 2020 until the end of the 2019/2020 academic year at the end of June 2020, all teaching and exams at the University of Burgos were solely conducted online. No rules on how teaching had to be performed were imposed, nor on how exams had to be held, nor on how to communicate with students, so each teacher was free to choose. It was found that some teachers opted for asynchronous teaching, while others preferred to teach classes in real time using online communication tools [38].
- Throughout Spain, classes were taught face-to-face during the 2020/2021 and 2021/2022 academic years (from September 2020 to June 2022), adopting strict safety measures: an interpersonal distance of 2 meters, mandatory use of face masks, continuous ventilation of classrooms, hand disinfection at the entrance to the classroom, and registration of class attendance through QR codes. Nevertheless, a hybrid-teaching-modality regulation was established for simultaneous online and face-to-face classes, so that students confined by contagion or close contact with infected persons could attend classes remotely, and so that the teaching could be given online when the teacher was confined [40]. Therefore, although online teaching was present throughout the study period, teaching was solely conducted online for around four months. The results may not therefore be applicable to teaching on Civil Engineering Degrees where the period of solely online teaching was considerably longer, which may be considered a limitation of the study.

### 2.2. Study development

The study was conducted in May 2022, at the end of the 2021/2022 academic year. A time period that was chosen because the teachers had experienced the three teaching phases described in relation to the COVID-19 pandemic (pre-pandemic, during lockdown, and post-lockdown). At that time, different teachers of the Bachelor’s Degree in Civil Engineering of the University of Burgos were contacted, and 12 of them, whose demographic characteristics are detailed in section 2.3, agreed to participate. Four meetings were held with those teachers, each lasting 40 minutes:

- The first three were discussion sessions in which the participating teachers were asked to reflect upon their teaching during each teaching stage of the COVID-19 pandemic [26]. The first three sessions covered the three phases, which offered the opportunity to the participating teachers to exchange experiences, to discuss the critical aspects of teaching in each phase, and to refresh their memories of their teaching during each phase. The sessions were moderated with an agenda of topics for discussion: general methodology used to teach the classes, changes in teaching methodology, student responses to the changes, communication with students…
- During the fourth session, the participating teachers were administered the survey, which is detailed in section 2.4. The plan was for all participating teachers to complete that survey at the same time, so that no teachers could complete the survey before the others and discuss their responses with those teachers who had not yet completed it, which might otherwise have influenced the results [17]. If necessary, doubts were resolved by moderators (the authors of the study).

The responses to these surveys were the results of the study, the analysis of which is presented in section 3, in accordance with the points detailed in section 2.5.

### 2.3. Participants

As indicated in section 2.2, a sample of 12 teachers was considered in this study, with an average age of 50.92±11.29 years. Other teacher-related educational studies conducted at the University of Burgos [41, 42] had similar sample sizes to this study. The sample of teachers was therefore thought to be representative of the staff teaching the Bachelor’s Degree in Civil Engineering at the University of Burgos. The participating teachers were responsible for teaching approximately 80 ECTS of the Bachelor’s Degree in Civil Engineering. The approach employed in the study is a valid way of investigating the lessons learned from the COVID-19 pandemic on the Bachelor’s Degree in Civil Engineering at small universities, such as the University of Burgos. Thus, the experience of teaching Civil Engineering Degrees at larger universities, if studied, might reveal other innovative approaches to teaching during the pandemic.

The participating teachers were teaching a wide range of courses, covering economics, project drafting, hydraulic calculation, structural calculation, business management, geotechnical engineering, building engineering, urban planning, environmental impact assessment, organization of civil works, prevention of occupational hazards, and the preparation of the final degree thesis. Laboratory technicians were not involved, even though the teaching of some courses was conducted in the laboratories, as classroom teaching according to Spanish educational regulations is an activity that only teachers can perform, and the technicians are simply there to assist.

All participating teachers, of legal age, gave their explicit written consent (email) to voluntarily participate in the study. No approval of this educational research by an ethics committee was required by the university regulations. It was enough to have the explicit consent of the participating teachers on a voluntary basis as long as their anonymity was always guaranteed in all publication stages of this study.

### 2.4. Instrument: Survey

The survey administered to the teachers participating in the study during the fourth session consisted of three open-ended questions to be answered within 40 minutes with no word limit for their response. These questions were posed in a general approach, seeking to detect common reflections from teachers on different civil-engineering teaching modules. Furthermore, the questions were of a progressive nature, so that each question addressed the teaching delivered during each teaching phase, in the following order: pre-pandemic, during lockdown, and post-lockdown. The survey was designed so that the participating teachers could progressively reflect upon the teaching during each phase and compare it with the teaching during the previous phases [20]. The development of the survey was based on previous studies of the authors [17, 20], whose experience with this type of research was complemented with other studies available elsewhere [41, 43]. The teachers were asked the three following questions:

1. What teaching methodology and tools did you use in your face-to-face teaching before the COVID-19 lockdown?
2. During the COVID-19 lockdown between March and May 2020, how did you adapt your teaching to conduct it completely online? Which aspects have improved or worsened compared to your previous teaching methods?
3. After the lockdown, in the 2020/2021 and 2021/2022 academic years, you returned to face-to-face teaching but with several sanitary restrictions. How was your teaching methodology during that time? How would you describe it? Has your teaching changed as a result of the lockdown experience?

### 2.5. Analysis of results

As the survey questions were open-ended, the answers were fragments of text, which underwent two different types of analysis using *Atlas*.*ti* software [44]:

- On the one hand, a qualitative analysis was performed with the aim of drawing general conclusions from the particular opinions of the teachers. The analysis began with a grouping and hierarchization of the text according to the ideas that were addressed. An operation that one of the authors of the study initially carried out, before it was subsequently reviewed by the rest of the authors. The same method was followed over several rounds, until no further modifications were introduced. In this way, continuous feedback and the shared viewpoints of all the authors of the study assisted with the grouping and hierarchization of the text fragments for their optimal organization [45].
- On the other hand, a mixed analysis based on word counting was completed, generating word clouds from the responses to each question. The conclusions of the qualitative analysis could therefore be corroborated, by observing the word clouds to identify the words that reoccurred most frequently.

## 3. Results

### 3.1. Qualitative analysis

#### 3.1.1. Teaching before COVID-19 lockdown

Before COVID-19 lockdown, the classes were face-to-face and mainly followed the traditional lecturing model, in which the teacher explains the theory and solves the exercises with no student participation [24]. The use of computer tools to support the presentation of theoretical concepts was widespread. A few teachers also mentioned that they provided digital media with explanations to some exercises, in addition to solutions presented on the blackboard during class time, so that students could refer to additional material on the exercises covered in class. Both the exercises and the solutions provided on digital media had almost always been solved in class, so students usually had no additional exercises without answers for home work.

*"Classes […] were exclusively face-to-face […]" "Classes focused on theory and problem-solving were presented with the help of PowerPoint […] and the blackboard." "[…] Lectures supported by PowerPoint presentations and multimedia resources […]" "Blackboard for solving exercises, supported by some exercises delivered in digital format […]"*

Despite the predominance of lectures, the utility of applying aspects addressed in class to real cases is also evident in the teaching of civil engineering [36]. Thus, different teachers emphasized that they applied each concept in the classroom to a real case or organized visits to construction sites, companies, and other places of interest. Many teachers also said that they organized practical classes outside the classroom in laboratories, and computer rooms where students could participate more directly by using software and testing machines. These sorts of classes brought the students closer to professional practice [18].

*"[…] I used to present real examples to the class […]" "Field visits were organized […] These used to be to company facilities or civil works that were in construction […]" "I often used the computer classrooms to explain some course-related software […]" "[…] practical classes were held in laboratories and workshops." "[…] in some courses, I had a large number of practical classes, which took place in the school workshops, with manipulation of material and equipment by the students."*

Teachers also indicated that during some of the class sessions they applied teaching methodologies that were intended to promote more active participation among students in their learning. On the one hand, some teachers indicated that they left time in class for students to solve exercises, trying to favor autonomous and cooperative learning, which allows students to develop interaction skills for professional practice [17]. The approach to professional practice is also favored in project-based learning [46], a methodology that some teachers also followed in the group projects that they requested. On the other hand, several teachers used the flipped classroom for the explanation of some theoretical concepts, so that students had to learn and to understand the concepts before the class [47].

*"For some exercises, I left time for students to work in class individually or in small groups before the full answer was presented on the blackboard." "[…] The creation of small groups (3 to 5 students) was frequent for the resolution of cases and problems […]" "[…] group work develops interaction skills useful in professional practice […]" "[…] group work was based on practical cases following the project-based learning methodology […]" "[…] some student presentations were in the classroom and were related to theory that had not yet been addressed, so that they could became broadly familiar with it, before it was explained in detail […]" "We also organized activities where the students explained the theory to their classmates […]"*

In relation to out-of-class communication with students, the teachers reported that it was mainly based on face-to-face meetings in their respective offices. However, it was also mentioned that email was very intensively used for the resolution of doubts, as it facilitates swift and flexible electronic communication [48].

*"The tutorials were held in person in my office […]" "[…] doubts were resolved by email many times […]" "The tutorials were conducted face-to-face, and many doubts were also resolved by email […]"*

Finally, in relation to exams, all the teachers reported that exams were always face-to-face, both the partial exams taken as part of the continuous evaluation and the official final exams, taken on the date and in the classroom officially established by the university.

*"Continuous-assessment exams were given in class […]" "[…] Individual final exams were held on dates and in the classrooms set by the university […]" "Individual final exams on both problem solving and theoretical issues were organized […]"*

#### 3.1.2. Teaching during COVID-19 lockdown

Teaching went completely online during the COVID-19 lockdown. Teachers basically employed two different teaching methods. Some opted to hold classes in real time using online videoconference tools such as Skype and Microsoft Teams. Others chose asynchronous teaching of theoretical aspects and problems through video recordings, which balanced work and family life [21]. These teaching methods were complemented by providing a greater amount of documentation on the platform, mainly problem solving, with the aim of increasing their autonomous learning [37]. Teachers also pointed out that practices and visits could not take place, although in some cases they could be replaced by activities that students could do at home.

*"Classes were taught via Skype." "[…] Teaching during that period was through Microsoft Teams […]" "[…] Online teaching made it necessary to use recorded videos […]" "Some theory classes went on to be taught by recorded video presentations […]" "For exercises, I recorded videos where the exercises were explained step by step […]" "[…] Students had the chance to watch a theoretical class again, or a recorded exercise they hadn’t understood. The information was always available." "The teaching platform became more prominent, all the information was uploaded […]" " […] I provided the students with material such as problems with solutions […]" "I uploaded additional videos for those who were interested and complementary material […]" "The visits and workshop practices could not be carried out […]" "[…] The practical workshop classes had to be replaced by new activities that they could do at home, such as analyzing the electrical and plumbing installations of their homes […]"*

Within this online-teaching context, teachers also indicated that interaction during class with students was greatly reduced. Teachers who taught their classes in real time using an online videoconference application mentioned that the students turned off the computer camera. In doing so, teachers were unable to perceive the non-verbal language of students to know whether the concepts had been well understood and if it was necessary to emphasize some aspects. This aspect is very important when giving master classes on problems and practical applications, so that teachers can adapt their explanations to the perceived levels of student understanding, the assessment of which also extends to body language [49]. This situation led many teachers to promote interaction with their students by asking questions during classes and using computer tools such as Kahoot, through which teachers and students can communicate in real time [50]. The teachers who opted for an asynchronous methodology also promoted this student-teacher interaction, launching questions on chats through the teaching platform, in which all the students were expected to participate.

*"In general, students had the camera turned off and participated less during the classes conducted via Microsoft Teams […]" "[…] I think it was more difficult for the student to maintain attention […]" "Information on whether students were understanding the concepts that were being explained was lost as no feedback was received from their non-verbal language […]" "To encourage participation I asked questions and looked for answers […]" "[…] I used Kahoot to make the class more participative and to know whether the students were following the class […]" "[…] In the case of the problems I used the resource of transferring the solved exercises and commenting on them with the students through a chat." "Some theory classes were taught by means of recorded video presentations that were later commented with the students in a chat on the teaching platform […]"*

Teachers also highlighted that the application of teaching methodologies that required peer interaction was more difficult to implement in online teaching. Some teachers thought that less interaction was a great loss, as classmates often explained theoretical concepts and helped with autonomous learning and solving exercises [30]. However, some teachers maintained the initial approach of group projects, organizing group videoconferences with students, whose success is linked to close monitoring by the teacher [31].

*"[…] With online teaching, interaction between students was lost […]" "[…] it was more difficult for them to work in small groups […]" "[…] the part where students explained theoretical aspects to their classmates or solved practical exercises within groups was lost." "[…] there was no chance for students to work individually or in groups on exercises in class before the solutions were presented […] these times are usually when students show a more active attitude […]" "The work was kept in a similar way to face-to-face teaching, students completed them in groups, but they met by videoconference […]"*

Out-of-class communication between the teacher and the students during the COVID-19 lockdown was also modified [23]. Many teachers opted for tutorials via Skype or Microsoft Teams, so contact could be maintained with the students. These meetings were also used to find out a little more about the personal situations of students and how they were coping with lockdown. Exchanges that favored closer contact between the teacher and the students than in the usual face-to-face classes. Despite the use of online tools, email continued to be the main method that the students used to send queries to the teachers, thanks to its immediacy and flexibility [48]. Finally, it should be noted that communication from the teachers to the students to give them feedback on their assignments and errors, and to inform them of the course-work and assignments each week and the most important aspects to study, were much more frequent.

*"[…] Skype tutorials encouraged close contact with students […]" "The tutorials were conducted using Microsoft Teams […]" "[…] every week I connected with several students via Microsoft Teams to discuss the work uploaded on the teaching platform and their doubts […]" "[…] the tutorials brought me closer to the students’ personal situations […]" " […] students discussed their personal situation more and that created a closer environment, with greater empathy and understanding […]" "For the most part, students continued to prefer tutoring via email […]" " I think the feedback provided to the students was much more intensive than during the face-to-face teaching […]" "[…] every week I sent them a personal email, breaking down the errors in their exercises […]"*

Finally, exams were conducted through the use of online questionnaires prepared on the teaching platform. Exams for the evaluation of practical concepts were in general identical to those used for classroom teaching but took place remotely. The adaptation of the students to this novel form of evaluation was successful.

*"The problem-solving exam was done in the same way as the face-to-face classes, but the students were connected to Microsoft Teams during the exam […]" "[…] the final exam of theoretical content was done through a questionnaire on the teaching platform […]" "For the exam, the questionnaire tool on the teaching platform was used […]" "There were no problems carrying out the exams and their revisions. The students quickly adapted to the new methodologies and tools […]"*

#### 3.1.3. Teaching after COVID-19 lockdown

During the 2020/2021 and 2021/2022 academic years, teaching on the University of Burgos Bachelor’s Degree in Civil Engineering was completely face-to-face. Therefore, the teachers reported that the teaching methodologies used before the COVID-19 outbreak had resumed. However, the situation had not completely returned to normal, because social-distancing was still an obligation that limited the possibility of students working in groups, which constrained their autonomous learning [17]. In addition, although the face-to-face teaching meant greater interaction with the students, the compulsory use of masks during class also meant that the teachers could not perceive the students’ facial expressions, which is otherwise very helpful to ascertain whether concepts have been properly understood [51].

*"My teaching is as it was before the pandemic […] I conduct similar lectures and let students solve exercises by themselves […]" "My current teaching is basically the same as pre-pandemic teaching […]" "[…] I have maintained the autonomous work of students and that in some classes students explain theory to their peers […]" "[…] the group projects I ask for follow a project-based learning approach, as before the pandemic […]" "Social distancing poses problems for group work in the classroom […]" "[…] I prefer the face-to-face class as a way of interacting with the student […]" "The use of face masks makes communication difficult as we cannot see anyone’s face […] we have to make a greater effort to encourage students to express their ideas and doubts"*.

It was also found that civil-engineering teachers introduced novel practices resulting from the pandemic-related restrictions, whose usefulness in civil engineering has not been previously verified in the literature:

- Virtual visits were introduced, both to companies and civil works. This practice provides the applied concepts with more direct applications to the professional world in straightforward logistical terms for both the teacher and the students [36].
- Other teachers mentioned that they increased interaction with students during face-to-face classes, asking them more questions and using real-time quiz tools such as Kahoot. The aim was both to establish their level of understanding of the concepts and to relieve classroom monotony [50].
- Many teachers also stated that after the COVID-19 lockdown they provided students with larger quantities of teaching material through the teaching platform, so that those students who wished to do so could work on the course autonomously. Other studies have shown that this is an aspect demanded by engineering students [20].
- Some teachers who always solved problems on the blackboard began to digitalize their solutions to provide them to students. In this way, students had at their disposal both the classroom solution and the one provided on the teaching platform, thus improving the understanding of the exercises [8].
- Finally, it was also noted that some of the students’ in-class presentations had been replaced by recorded videos. The idea was that these videos should always be available to the whole class for viewing and that they could form the basis for brief exams and continuous evaluation questionnaires.

*"[…] now I also make videoconferences with companies in the sector or virtual visits to construction sites […]" "To try to find out the student’s level of understanding, I question the students more than before during the classes […]" "[…] I have incorporated the use of Kahoot in class […]" "[…] I have included more resources and materials in the teaching platform […]" "[…] I have made more supplementary material available to students in the teaching platform […]" "I have digitalized the solution of many exercises […] so that students do not only have their solution on the blackboard […]" "I provide students with the answers to the exercises solved in class on computer media […]" "Now students do audio-recorded presentations on their PowerPoint presentations for some of the assignments […] so their classmates can view it several times and I can ask about it in continuous-assessment tests."*

With regard to out-of-class communication between the teacher and the students, email continued to be widely used, but online videoconferencing was maintained and promoted by the teachers, due to its simplicity, speed of communication, and temporal and spatial flexibility [22].

*"If the student so requests, tutoring or exam reviews are virtual […]" "[…] nowadays there is the possibility of tutoring via Microsoft Teams, which sometimes makes it easier for students." "[…] I am more open to distance options, such as tutoring by videoconference […]" "[…] tutoring by videoconference is much more versatile than face-to-face tutoring […] it’s easier to organize a session at any time […]"*

Finally, a few teachers mentioned that they were considering continuous-assessment exams using computer tools and questionnaires. They considered that these sorts of tools would be ideal for setting short exams with spatial and temporal flexibility, which would also facilitate the preparation for the final exam.

*“I am considering using questionnaires from the teaching platform for some continuous-assessment exams […]” "The continuous-assessment exams are short […] computer tools could be used to make it easier for students to take the exams and to assist with final exam preparation."*

### 3.2. Word-counting mixed analysis

**Fig 1:**
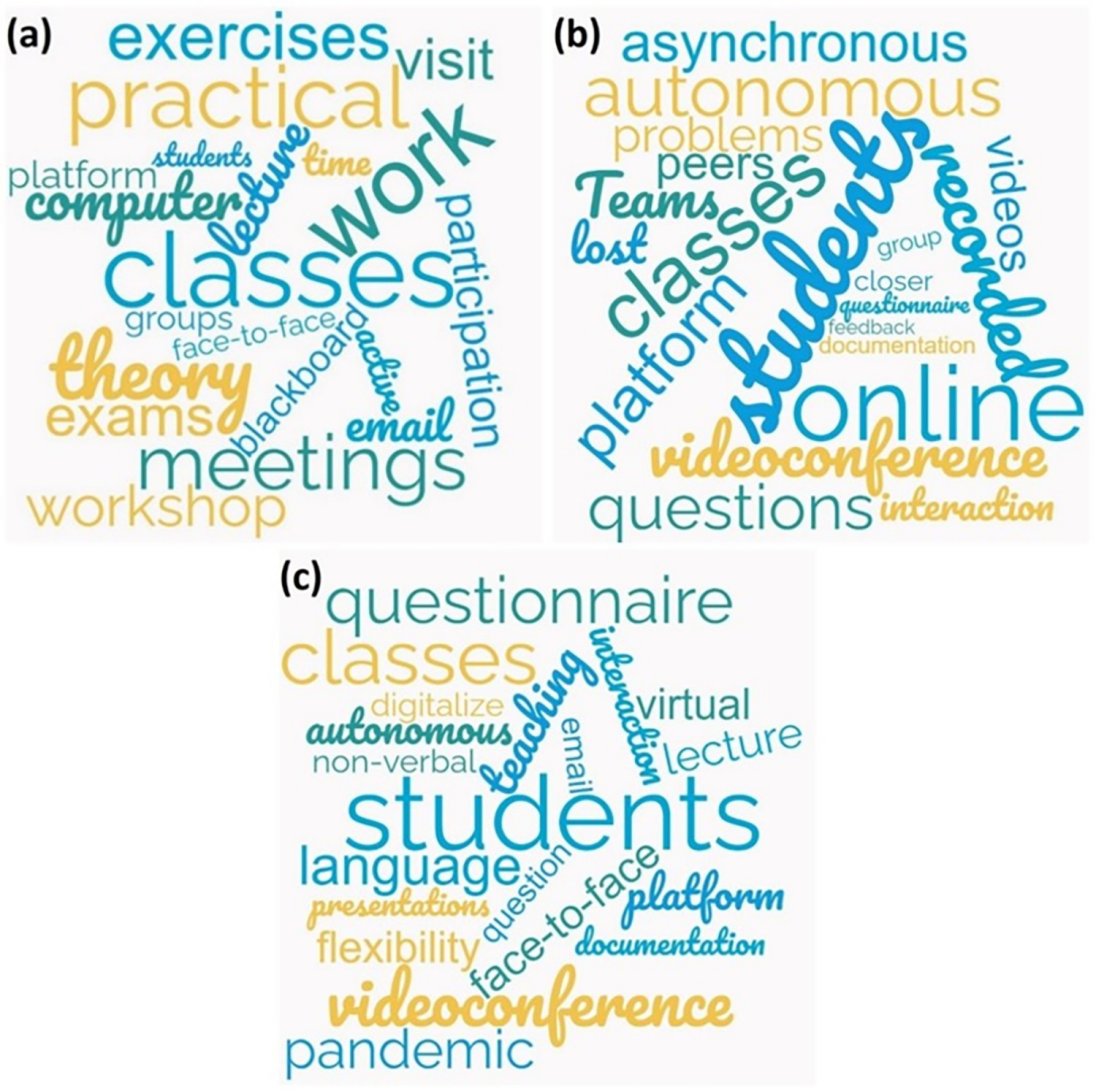
Word clouds of the teachers’ answers: (a) pre-COVID-19 pandemic and lockdown; (b) during COVID-19 lockdown; (c) post-COVID-19 lockdown.

A word-counting mixed analysis of the responses from the teachers to each of the three questions was performed for verification of the qualitative analysis and its conclusions. The 20 most repeated teaching-related words in the responses are represented in the word clouds shown in Fig 1.

The word cloud for the answers to the question on the characteristics of pre-pandemic civil-engineering teaching is depicted in Fig 1A. It shows that most of the *“classes”* were taught through *“lectures”* supported by *“computer”* applications for the *“theory”* and the resolution of *“exercises”* on the *“blackboard”*, the teachers providing the documentation on the teaching *“platform”*. Site “*visits”* and *“workshop”* practices were also common to provide a *“practical”* application to the aspects explained in class. In addition, words related to teaching methodologies that try to promote a more active implication of *“students”* could also be found, such as *"groups"*, *“work”*, *"active"*, and *"participation"*. Finally, it should also be noted that the words *"email"*, *“time”*, *"meetings"*, *"exams"*, and *"face-to-face"* frequently reoccurred, clearly showing the main ways of conducting exams and student-teacher out-of-class communication.

The characteristics of civil-engineering teaching during the COVID-19 lockdown outlined above also coincide with the aspects derived from Fig 1B. The abrupt move to *“online”* teaching based on *“recorded” “videos”*, *“asynchronous”* presentations, *“classes”* through online *“videoconference”* tools such as Microsoft *“Teams”* and the search for more *“autonomous”* work among *“students”*, mainly regarding *“problems”*, by providing them with a greater amount of *“documentation”* through the teaching *“platform”* can be appreciated. Words such as *"lost"*, *"interaction"*, *"questions"*, *“group”* and *"peers"* can also be distinguished, which show that peer work and student-teacher interaction were lost during lockdown time. The out-of-class student-teacher relationship was *“closer”*, due to the health situation and to the more personalized *“feedback”* from teachers on the work of their students. Online *“questionnaires”* for exams were also common.

The most repeated words regarding teaching after COVID-19 lockdown are shown in Fig 1C. Therefore, *“face-to-face” “classes”* were resumed through *“lectures”*, also applying methodologies for students’ *“autonomous”* learning. However, a series of changes in *“teaching”* were detected in relation to before the COVID-19 *“pandemic”*: teachers attached greater importance to *“interaction”* with *“students”* and *“non-verbal” “language”*, asking more *“questions”* to check class follow-up; *“virtual”* visits were made to construction sites and companies; problem solutions began to be *“digitalized”* to provide them through the *“platform”*, as well as a greater amount of *“documentation”*; students delivered some audio-recorded *“presentations”*; out-of-class communication was promoted through *“email”* and *“videoconferences”*, due to the *“flexibility”* they offered; and some short exams were administered through online *“questionnaires”*.

## 4. Overall discussion

**Fig 2:**
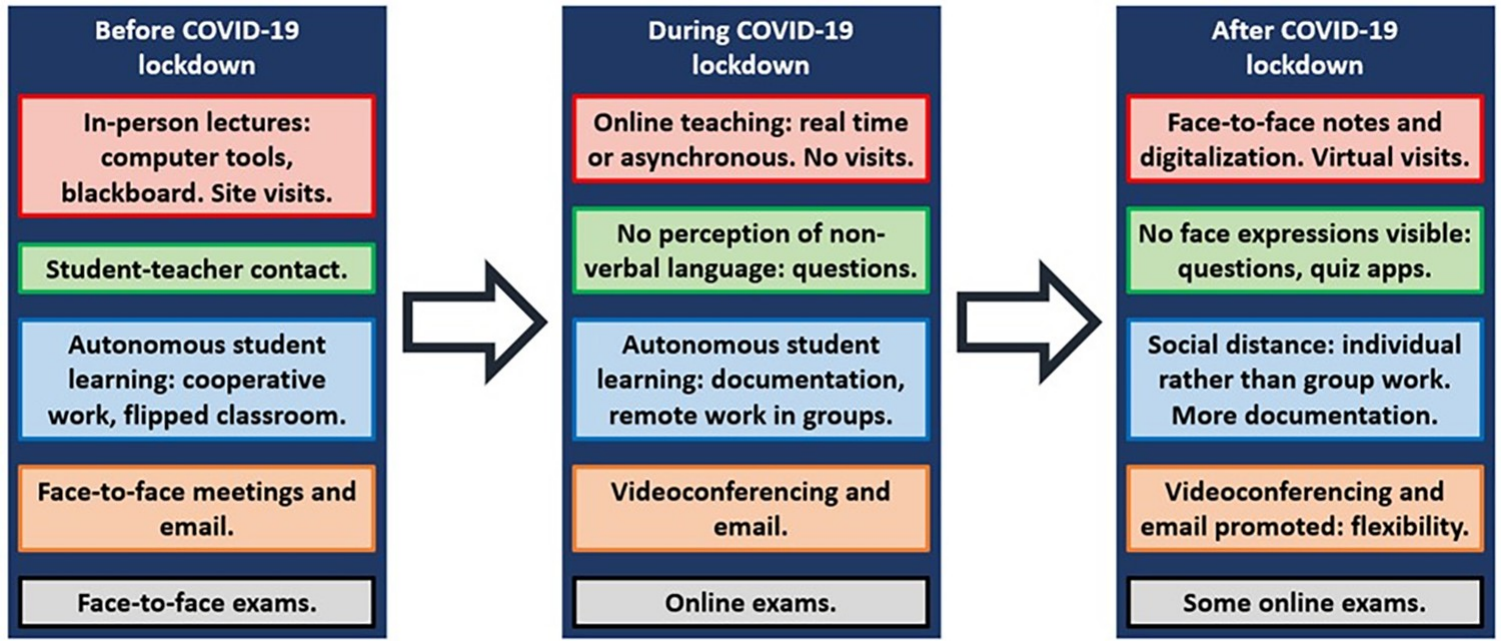
Overview of civil-engineering teaching during COVID-19 pandemic.

From the results of faculty staff interviews on the characteristics of civil-engineering teaching during each teaching stage of the COVID-19 pandemic (pre-pandemic, during lockdown, and post-lockdown), an overview of civil-engineering teaching during this period of time can be constructed, as shown in Fig 2.

Prior to the COVID-19 outbreak, teaching in civil engineering at the University of Burgos, Spain, was mainly characterized by lectures imparted with the support of computer software programs and the blackboard for solving exercises. Site visits and workshop practices were also commonplace to show real applications of the aspects covered in class [27]. Furthermore, the teacher could perceive the non-verbal language and facial expression of the students, and could ask them direct questions, to find out whether the concepts had been properly understood. The variety and complexity of concepts addressed in civil-engineering courses means that direct contact with students is essential for the teacher to have a clear idea of the level of learning [34]. Innovative teaching methodologies, such as individual autonomous work, cooperative work, flipped classroom for theoretical concepts, and project-based learning were also used. These methodologies sought a more active and autonomous attitude of students in their learning [52]. Exams and tutorials were face-to-face, although many students used email for setting down their doubts, due to its speed and flexibility [48].

The COVID-19 outbreak and lockdown caused a sudden change from face-to-face lectures to online teaching, either in real time through online videoconference tools such as Microsoft Teams, or asynchronously through recorded videos. The closer contact between teachers and students led students to assess the civil-engineering teaching received during this time positively [38]. However, the teachers highlighted negative aspects, such as the fact that site visits could not be conducted, and that trying to promote students’ autonomous work was more complicated, as they could not be assigned time in class for individual or group work. Thus, many teachers opted to increase the documentation available on the teaching platform, so that students could work more on their own [53], and to organize remote group meetings, which has proved quite acceptable when the sessions are properly moderated [31]. Despite all this, the main negative aspect for the teachers was the lack of direct interaction with the students, either because of asynchronous teaching or because the students turned off the camera during the real-time classes. It meant that the teachers could not perceive the non-verbal language and the facial expressions of the students, which are often indicative of whether a concept has been properly understood [14]. Therefore, some teachers decided to increase the number of questions they asked students in class, or to use real-time quiz tools such as Kahoot and forums on the teaching platform. The aim of these three strategies was to determine the level of understanding, and to promote a more active attitude among students [50]. The tutorials were mostly conducted through videoconferences and email. The exams were conducted through online questionnaires.

After the COVID-19 lockdown, in the 2020/2021 and 2021/2022 academic years, face-to-face teaching in civil engineering was restored. Thus, the traditional lectures were recovered, but the teaching methodologies that required group work among students within the classroom could not be implemented, due to the compulsory social distancing. Faced with this situation, the teachers promoted not only individual autonomous work, of proven effectiveness on technical courses [37], during their classes, but they also provided more documentation on the teaching platform and digitalized the solution of the exercises. Their idea was to provide students with teaching material to work on outside the classroom [53]. For the same reason, it was not possible to visit companies and construction sites, which was compensated by virtual visits, classroom presentations of real examples, and student deliveries of audio-recorded presentations so that all classmates could see and evaluate them, thereby raising interest levels and involvement in the work of their peers [30]. The other major difference with traditional teaching was the obligatory wearing of face masks, which limited the teacher’s perception of students’ non-verbal language and facial expression. Therefore, teachers asked more questions to the students and assiduously employed real-time quiz tools not only to enliven the class, but also to determine which concepts to emphasize over others [54]. The effect of COVID-19 lockdown could also be perceived in the out-of-class communication and in the exams. Thus, the use of online videoconference tools was promoted, a means of communication that was successful when the tools were adapted to the type of concept addressed [23]. In addition, some teachers were open to the idea of setting short exams through online questionnaires to promote final exam preparation. The experiences on civil-engineering teaching derived from the COVID-19 pandemic are a step forward to try to improve and to modernize teaching in this engineering field at small universities, such as the University of Burgos.

## 5. Conclusions

In this study, the characteristic features of teaching on the Bachelor’s Degree in Civil Engineering at the University of Burgos, Spain, pre-pandemic, during the COVID-19 pandemic, and post-pandemic have been analyzed. To do so, teachers giving several courses on this Bachelor’s Degree were invited to reflect on their teaching in each of the three teaching stages during the COVID-19 pandemic by answering a survey. A generic approach was adopted in the survey, with an open question referring to each teaching stage, so that the study would be valid for courses related to different civil-engineering disciplines. The qualitative and mixed analysis of the teachers’ answers to the survey yielded the following conclusions on the aspects affected more than any other during the COVID-19 pandemic and the changes that it meant for teaching on the Bachelor’s Degree in Civil Engineering.

- Interaction with the students, as well as the analysis of their non-verbal language and facial expression, are fundamental to determine the level of understanding of the concepts. Frequent questions during the class and the use of real-time quiz tools, such as Kahoot, not only enlivened the classes, but also helped to determine which concepts should be emphasized the most, in order to guarantee an adequate follow-up of the course.
- The autonomous learning of students should be promoted with teaching methodologies to be applied in class, such as cooperative group work, autonomous individual work and flipped classroom experiences. However, it may also be useful to provide additional documentation, such as extra exercises, and to digitalize the solutions to the exercises covered in the classroom, so that students who wished to do so could work on their own, deepening their knowledge of the course work.
- Visits and workshop practices should be promoted to show the practical application of the concepts studied and to bring students closer to the professional world. However, interspersing these aspects during the explanation of the concepts in class can further promote their understanding. Hence, the utility of virtual visits and real visual examples.
- New forms of delivering assignments can be encouraged, such as audio-recorded presentations instead of oral presentations in class. The objective is for the students to produce useful projects, both for themselves and for their classmates. Through audio-recorded presentations, all students could see the work of their peers as many times as necessary, and could study and analyze its most important aspects. The concepts that were addressed on those presentations could therefore be the subject of a more general evaluation.
- Online videoconference tutorials can be an interesting option to achieve greater immediacy in communication with students and to provide greater spatial and temporal flexibility. To do so, they should be properly adapted to the type of theoretical or practical concept that is presented.
- Finally, administering mainly continuous-assessment exams online is a practice that can be further explored. Giving considerable flexibility to the teacher, these sorts of exams are often preferred among students, often leading to better student learning and final exam preparation.

The teachers of the Bachelor’s Degree in Civil Engineering at the University of Burgos, Spain, have learnt the six lessons described above during the COVID-19 pandemic and have shared them here as teaching innovations and to modify the conventional patterns of teaching in civil engineering, seeking to encourage active attitudes and to achieve maximum levels of understanding and learning among students. A subsequent step might be to assess whether these lessons learned are also found in larger universities or universities that had longer online teaching periods, which are among the main limitations of this study. Furthermore, measuring the implementation level of these six aspects when the COVID-19 pandemic is eventually declared over might also be a relevant area for future research.
